# Untangling SNP Variations within *CYP2D6* Gene in Croatian Roma

**DOI:** 10.3390/jpm12030374

**Published:** 2022-02-28

**Authors:** Anita Stojanović Marković, Matea Zajc Petranović, Željka Tomas, Borna Puljko, Maja Šetinc, Tatjana Škarić-Jurić, Marijana Peričić Salihović

**Affiliations:** 1 Institute for Anthropological Research, 10000 Zagreb, Croatia; astojanovic@inantro.hr (A.S.M.); matea@inantro.hr (M.Z.P.); maja.setinc@inantro.hr (M.Š.); tanja@inantro.hr (T.Š.-J.); 2 Department for Translational Medicine, Srebrnjak Children’s Hospital, 10000 Zagreb, Croatia; ztomas@bolnica-srebrnjak.hr; 3 Croatian Institute for Brain Research, School of Medicine, University of Zagreb, 10000 Zagreb, Croatia; borna.puljko@mef.hr; 4 Department for Chemistry and Biochemistry, School of Medicine, University of Zagreb, 10000 Zagreb, Croatia

**Keywords:** *CYP2D6*, ADME, pharmacogenetics, population genetics, star allele, Roma, Croatia

## Abstract

*CYP2D6* is a highly polymorphic gene whose variations affect its enzyme activity. To assess whether the specific population history of Roma, characterized by constant migrations and endogamy, influenced the distribution of alleles and thus phenotypes, the *CYP2D6* gene was sequenced using NGS (Next Generation Sequencing) method-targeted sequencing in three groups of Croatian Roma (*N* = 323) and results were compared to European and Asian populations. Identified single nucleotide polymorphisms (SNPs) were used to reconstruct haplotypes, which were translated into the star-allele nomenclature and later into phenotypes. A total of 43 polymorphic SNPs were identified. The three Roma groups differed significantly in the frequency of alleles of polymorphisms 6769 A > G, 6089 G > A, and 5264 A > G (*p* < 0.01), as well as in the prevalence of the five most represented star alleles: *1, *2, *4, *10, and *41 (*p* < 0.0001). Croatian Roma differ from the European and Asian populations in the accumulation of globally rare SNPs (6089 G > A, 4589 C > T, 4622 G > C, 7490 T > C). Our results also show that demographic history influences SNP variations in the Roma population. The three socio-culturally different Roma groups studied differ significantly in the distribution of star alleles, which confirms the importance of a separate study of different Roma groups.

## 1. Introduction

The *CYP2D6* gene encodes the phase I drug-metabolizing homonymous enzyme and is located in tandem with pseudogenes *CYP2D7P* and *CYP2D8P* on chromosome 22q13.1, at the 3′ end of the *CYP2D* cluster [1,2]. It contains nine exons consisting of 1461 codons and is highly polymorphic, with more than a hundred genetic variations and numerous subvariants that differ in single nucleotide polymorphisms (SNPs) or copy number variations (CNV); the latter resulting from *CYP2D6* gene deletion or multiplication. In 1996, a group of international experts in pharmacogenetics decided to systematize allelic variants of *CYP2D6* proposing a haplotype-based star (*) nomenclature system [3]. Since then, mostly due to the development of DNA sequencing technology, a tremendous amount of allelic and suballelic variants have been identified and classified by the Pharmacogene Variation Consortium [4].

The most important consequence of *CYP2D6* genetic variations is their influence on the metabolizing activity of the CYP2D6 enzyme. These variations were broadly grouped into four different drug-metabolic phenotypes of the CYP2D6 enzyme; (1) poor metabolizer (PM—only null activity alleles detected), (2) intermediate metabolizer (IM—one normal activity allele with one null activity allele; or two decreased activity alleles), (3) extensive metabolizer (EM—two normal activity alleles; or a combination of one increased activity allele with one decreased activity allele), and (4) ultra-rapid metabolizer (UM—a combination of one normal activity allele with one increased activity allele) [5,6,7,8]. Metabolizing activity depends not only on the genotype, but is also influenced by a number of physiological, pathological, and environmental factors [9]. CYP2D6 is involved in the metabolism of up to 25% of drugs commonly used in medicine [10], although it constitutes only 2–4% of the total CYP content in the liver [11,12]. CYP2D6 metabolizes a wide range of drugs: antiarrhythmics, tricyclic and second-generation antidepressants, β-blockers, anti-cancer drugs, several opioid analgesics including codeine and tramadol, and many more [13,14,15,16,17,18]. Additionally, variations in the *CYP2D6* gene have been studied as a risk factor for a number of diseases: Parkinson’s disease [19,20,21], schizophrenia and other psychiatric diseases [15,22], Alzheimer’s disease [23,24], and several forms of cancer [25,26].

The role of CYP2D6 in the metabolism of natural xenobiotics has been studied scarcely, but this enzyme is known to have a very high affinity for alkaloids [27]. Therefore, its evolutionary role is thought to be related to alkaloid metabolism in food. There is a hypothesis that due to food constraints relative to the size of the population in Northwest Africa some 10,000–20,000 years ago, a selection pressure occurred that favored the survival of subjects capable of detoxifying plant toxins to a higher extent, increasing the number of plants that can provide useful food [28]. Changes in diet throughout human history have exerted selective pressure on genes whose products metabolize food compounds, the best example being N-acetyltransferase 2 (e.g., [29]). Fuselli (2010) suggested that current patterns of genetic diversity in *CYP2D6* are the result of selective pressure imposed by new or more concentrated CYP2D6 substrates that emerged in food production especially at the beginning of the Neolithic transition, in the presence of poorer nutritional conditions and higher disease burdens [30].

Genes encoding phase I and phase II metabolizing enzymes, drug transporters, and modifiers show a population-specific diversity, indicating that their variability has been shaped by evolutionary mechanisms [31]. The global distribution of *CYP2D6* allele frequencies is differing. Some alleles are present at similar frequencies across the world, while others are ethnically or geographically specific. The nonfunctional allele CYP2D6*4 is the most frequently found in Europe, the decreased-function CYP2D6*10 allele is mostly present in Asia and East Asia, alleles CYP2D6*17 and *29 are characteristic for Africa and Afro-descendant populations, while CYP2D6*41 allele and gene amplifications are the most common in Middle Eastern populations [4,32,33]. Despite the proven functional role of CYP2D6 enzyme and its extensive research worldwide, knowledge of *CYP2D6* allele frequencies within isolated populations like the Roma population, Basques, Ashkenazi Jews, and Saami is very limited [34].

The Roma (Gypsy) population is a transnational minority present in many countries of the world. They originated in India and arrived in Europe around the 11th century via central Asia (Afghanistan and Persia), the Middle East, and present-day Turkey. The Roma population is estimated at 15 million people, of whom 12 million live in Europe. Roma in Croatia belong to two socio-culturally and linguistically different groups: Vlax Roma, who speak ljimb’d bayash language, and Balkan Roma, who speak dialects of romani chib language. The Vlax Roma are descendants of the Roma who crossed the Danube River between the 13th and 15th century and upon arrival in Wallachia, Transylvania (both in present-day Romania) and Moldavia were enslaved to work in the mines for the next 500 years. During that time, they were forbidden to use their own language, so their descendants are recognized by a specific archaic Romanian language. In the 19th century, after slavery was abolished, the Vlax Roma started a new migration wave; after leaving Romania, they settled in Hungary, Croatia, Serbia, and other Balkan states, as well as in other parts of Europe, while some even reached the United States [35,36]. Balkan Roma in Croatia are descendants of the Roma who arrived in the Balkans in the 11th century, and during the Ottoman expansion, some groups of these Balkan Roma moved further west. Socio-cultural characteristics of the Roma population, such as strict rules of endogamy, have led to the founder and the bottleneck effects that have caused a genetic structure of Roma to differ compared to other populations [35,37,38], which has been shown to affect ADME (Absorption, Distribution, Metabolism and Excretion) genes variations as well [39]. In Croatia, the Vlax Roma mostly inhabit the Baranja Region, in the eastern part of Croatia, and the Medjimurje Region, in the north of Croatia. The Balkan Roma mostly live in Zagreb area.

The main objective of this study was to estimate variation within the *CYP2D6* gene among three socio-culturally and geographically distinct Croatian Roma groups (Balkan Roma, and Vlax Roma from the Baranja region and Medjimurje region), to find out whether their specific population history influenced the distribution of *CYP2D6* alleles and consequently phenotypes.

## 2. Materials and Methods

**Figure 1:**
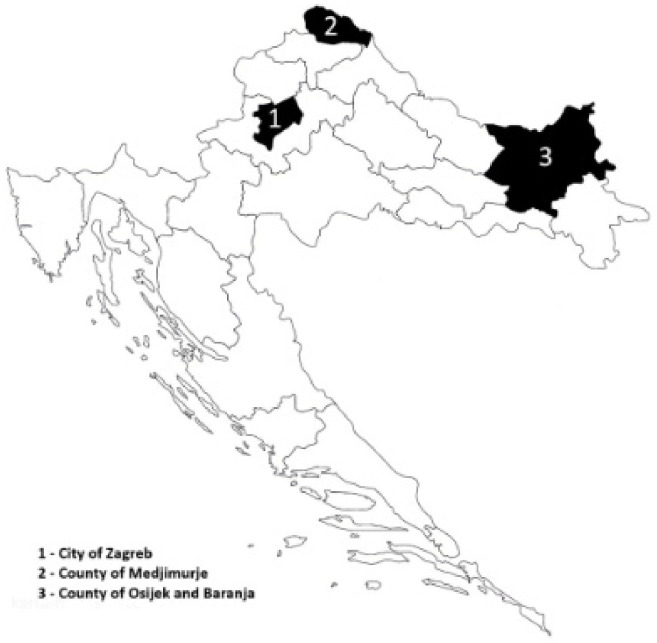
Sampling locations in Croatia.

We analyzed 323 DNA samples, all collected during field studies of the ongoing multidisciplinary anthropological, molecular-genetic, and epidemiological investigations of Roma groups in Croatia. Samples were collected during field research in Vlax Roma settlements in the Baranja region and Medjimurje region and in Balkan Roma settlements in the city of Zagreb, Croatia (Figure 1). All respondents participated in the research voluntarily, and with the help of Roma volunteers, were introduced to the objectives, methods, and the anticipated contribution of the project. The protocol of the study was approved by the Scientific Board and the Ethics Committee of the Institute for Anthropological Research in Zagreb, Croatia.

DNA was extracted from peripheral blood using the salting-out method [40]. The entire *CYP2D6* gene (ENSG00000100197 chromosome 22: 42,126,499–42,130,881, GRCh38.p12) was sequenced with Illumina MiSeq V3 kit using the method Genotyping-in-Thousands by sequencing (GT-seq) in a commercial laboratory. GT-seq is a multiplexed amplicon sequencing method that allows for the simultaneous genotyping of hundreds of SNPs across thousands of individuals in a single library, making library preparation simple and cost-effective [41]. Raw reads were demultiplexed and sequencing adapter remnants were clipped from all reads (reads with final length < 100 bases were discarded). Primer sequences were removed, and sequence fragments were turned into forward-reverse primer orientation. Low-quality reads were discarded (Phred quality score of 20 over a window of 10 bases). Quality trimmed reads were aligned against all clusters using BWA version 0.7.12. The FreeBayes v1.02-16 was used for variant discovery and genotyping of the samples. Variants were filtered using a set of GT-seq specific rules (minimum allele count must exceed eight reads, minimum allele frequency across all samples must exceed 10%).

Allele and genotype frequencies and Hardy–Weinberg equilibrium were calculated using VCFtools [42]. Differences in genotype and allele frequencies between Roma groups were tested using the Chi-square test and Fisher’s exact test. The analyses were performed using the SPSS Statistics 21.0 statistical package for Windows (SPSS Inc., Chicago, IL, USA) and *p*-values were corrected for multiple testing using Bonferroni correction.

*CYP2D6* gene sequencing identified 43 single nucleotide polymorphisms (SNPs), 28 of which were used for haplotype reconstruction. The 14 SNPs were excluded due to low level of heterozygosity (rs28371732, rs867985262, rs79596243, rs28578778, rs141009491, rs762158210, rs35742686, rs28371717, rs1349481801, rs376056664, rs189736703, rs28371703, rs1080992, and rs769811346). The low level of heterozygosity was considered for cases in which there was no minor allele in two Croatian Roma groups, while in the third Roma group it occurred with a frequency of two or less. The remaining insertion polymorphism (rs1269631565) was excluded because it cannot be phased by Phase software due to its indel nature. However, this polymorphism is not among star alleles’ defining SNPs.

Haplotypes of the Croatian Roma groups were inferred using Phase ver. 2.1 [43,44]. Haplotypes were translated into the star allele nomenclature using the data provided at the PharmVar website [45]. Star diplotypes were translated to phenotypes and classified into three metabolizer categories—normal, intermediate, and poor according to the guidelines [46].

Software Arlequin 3.5 [47] was used to infer intra-population diversity indexes (haplotype and nucleotide diversity) and population pairwise F_ST_ values, statistical significance assessed by generating 100,000 random samples. Possible departure from selective neutrality was tested using the Ewens–Watterson (EW) homozygosity test also implemented in Arlequin. Statistical significance was assessed by generating 10,000 random samples under the null conditions of no selection and constant population size.

The relations among haplotypes were shown in networks, which were calculated by the median-joining (MJ) procedure with default settings [48] using the program NETWORK 10.2.0.0 [49].

The inter-population comparisons of the Roma with other populations, based on frequencies of minor alleles, were performed using the data from the gnomAD database (v3.1.2) for East Asian, South Asian, and European (non-Finnish) populations. A comparison of the star allele distribution between the surveyed Roma groups and world population divided into major ethnic groups was performed using data from studies listed in Supplementary Table S2 in the work of Gaedigk (2017) [4]. Data were visualized in six graphs, each one representing a star allele found in more than 5% of the Roma in the Croatian Roma groups. Because the major ethnic groups consisted of data from multiple studies, the average star allele frequency for each major ethnicity group was calculated by weighting according to the sample size of each study.

## 3. Results

Sequencing of the *CYP2D6* gene in DNA samples obtained from three Croatian Roma groups (323 persons in total) reveal 43 polymorphic positions, which are listed in Table 1. Due to low heterozygosity (absence of heterozygotes and recessive homozygotes), only 21 SNPs could be tested for HWE in all three Croatian Roma groups. The remaining 22 SNPs were tested for HWE in only one or two Croatian Roma groups. Most of the SNPs which were polymorphic in all three Roma groups were in Hardy–Weinberg equilibrium (HWE); after applying Bonferroni correction, exceptions were intron variants 8602 A > G (rs2004511) in total Roma sample, 6188 G > A (rs1081004) in all three Roma groups and in the total Roma sample and noncoding transcript exon 5264 A > G (rs29001678) in total Roma sample.

A total of 21 SNPs were polymorphic in all three Roma groups (Table 1). Chi-square test results showed that four SNPs differed significantly in frequencies of genotypes between the three analyzed groups after Bonferroni’s correction: 8565 dup (rs1269631565), 6769 A > G (rs1135824), 6089 G > A (rs368389952), and 5264 A > G (rs1081000). A significant difference in allele frequencies between the three Croatian Roma groups was found in the same SNPs (after Bonferroni’s correction): 8565 dup (rs1269631565), 6769 A > G (rs1135824), 6089 G > A (rs368389952), and 5264 A > G (rs1081000).

**Figure 2:**
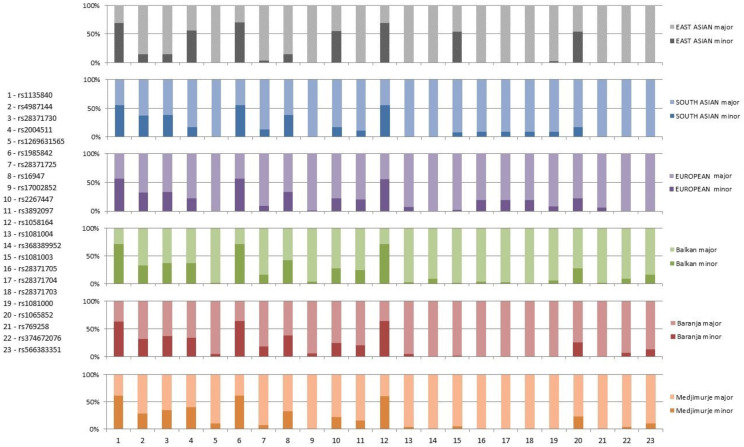
Allele frequencies of *CYP2D6* SNPs in East Asian, South Asian, and European (non-Finnish) populations (as reported in gnomAD database, version 3.1.1) and in three Croatian Roma populations.

Furthermore, we compared minor allele frequencies of 43 polymorphic SNPs from this study with their frequencies in East Asian, South Asian, and European populations. The lowest number of polymorphic SNPs was found in East Asian populations, where 18 out of 43 investigated SNPs were monomorphic and 13 SNPs had MAFs less than 1%. In South Asian populations five SNPs were monomorphic and six SNPs had MAFs less than 1%, while only two SNPs were monomorphic in Europeans, but 16 SNPs had MAFs less than 1% (Supplementary Table S1). SNPs found to be polymorphic in the investigated Roma groups had higher MAFs in South Asian than in the European populations. The distribution of allele frequencies of SNPs with MAFs > 0.05 in at least one of the populations is shown in Figure 2.

A total of 93 *CYP2D6* haplotypes were reconstructed from polymorphic SNPs, as explained in the Materials and Methods section (Supplementary Table S2). Intra-population analysis based on all reconstructed haplotypes revealed the highest diversity in the population of Roma from Baranja and the lowest among Roma from Medjimurje (Table 2). Pairwise population F_ST_ distances showed the highest difference between Balkan and Medjimurje Roma (F_ST_ = 0.01249), followed by similar distances between Roma from Balkan and Baranja (F_ST_ = 0.0028) and between Baranja and Medjimurje (F_ST_ = 0.0027). The exact test of sample differentiation based on haplotype frequencies showed significant differences between samples. In order to exclude the possible influence of selection on *CYP2D6* haplotype distribution, the Ewens–Watterson test of selective neutrality was performed. All Roma groups have higher observed than expected homozygosity, but results were insignificant which rules out the influence of directional selection.

A total of 89 haplotypes were translated into star nomenclature, which resulted in 10 star alleles (*) (Table 3). The reference CYP2D6*1 allele was the most common in each of the three Roma groups individually, and in the entire Roma sample (33.1%). In addition to *1, the other most prevalent alleles (total sample prevalence larger than 5%) were star alleles *2, *4, *10, and *41—the five listed star alleles were found in 96.4% of the total Roma sample. Of the five remaining star alleles, two were not found in all three Roma groups, and the CYP2D6*65 allele was found in only one person from Baranja.

Chi-square test results showed that all three Roma groups significantly differ amongst themselves in the prevalence of the five most prevalent *CYP2D6* star alleles (χ^2^ = 34.996, *p* < 0.0001) (Table 3). Comparing the three groups pairwise, results showed that Medjimurje Roma differ significantly from Balkan (χ^2^ = 24.759, *p* < 0.0001) and Baranja Roma (χ^2^ = 22.329, *p* < 0.0001), while Balkan and Baranja Roma do not (*p* = ns). We also compared the distribution of the five most frequent star alleles in the three Roma groups by comparing the prevalence of one star allele vs. other four merged ones; the distribution of four of them differs significantly among the three Roma groups (*1 − χ^2^ = 6.2788, *2 − χ^2^ = 6.2276, *10 − χ^2^ = 8.6023, *41 − χ^2^ = 16.1097; all have *p* < 0.05) (Table 3), while the distribution of *4 allele did not differ.

**Figure 3:**
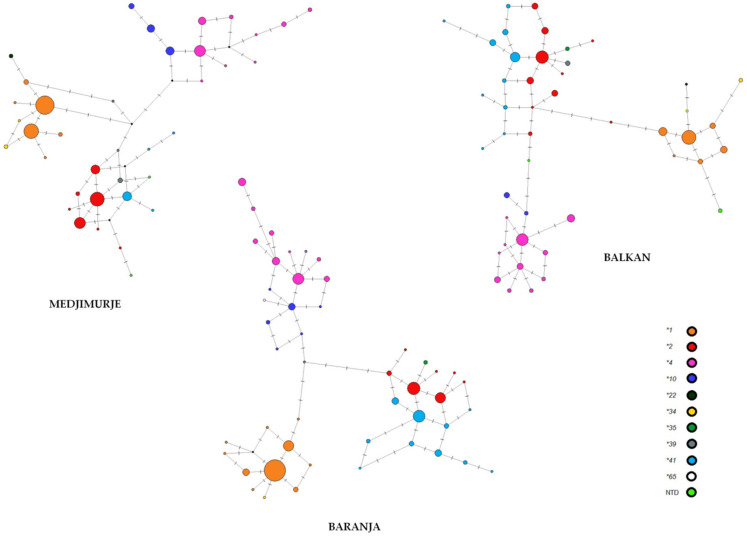
Median-joining networks of *CYP2D6* haplotypes in Roma from Balkan, Baranja, and Medjimurje. Haplotypes belonging to the same star allele are shown in a separate color. Distance between nodes is proportional to the numbers of SNPs whose alleles differ. SNPs are represented with lines. Size of nodes is an approximation of haplotype frequency. Black nodes represent phylogenetically possible haplotypes not found in this study. (NDT—not determined).

In addition, MJ networks were constructed to show the potential phylogenetic relationships among star alleles and their diversity in the studied Roma groups. All three MJ networks showed three clusters: one with highly predominant suballeles of *1, the other with suballeles of *2 and *41, and the last with suballeles of *10 and *4, suggesting that alleles *2 and *41, as well as the alleles *10 and *4, are phylogenetically close. Suballeles of *22 and *34 cluster together with suballeles of *1, suballeles of *35 cluster with suballeles of *2, while suballeles of *39 take an intermediate position between clusters in Roma groups from Baranja and Medjimurje. In Roma group from Balkans, the suballeles of *39 are placed among suballeles of *2. Suballele of *65, present in the Roma group from Baranja, is placed among suballeles of *10 (Figure 3). Six newly found haplotypes could not be translated into star alleles but according to the position in the MJ networks their classification to the star allele nomenclature could be estimated (Figure 3).

**Figure 4:**
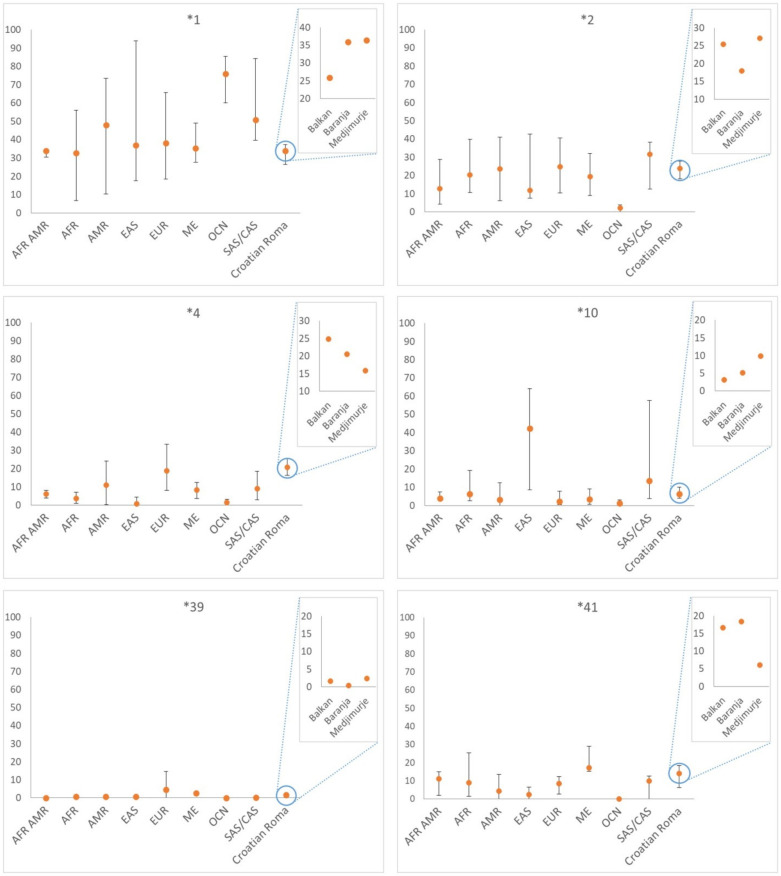
Distribution of *1, *2, *4, *10, *39, *41 alleles frequencies in Croatian Roma and major human populations groups (abbreviations: AFR AMR—African Americans, AFR—Africa, AMR—Americas, OCN—Oceania, EAS—East Asia, SAS/CAS—South/Central Asia, ME—Middle East, EUR—Europe).

We also compared the frequencies of alleles *1, *2, *4, *10, *39, and *41, estimated in the Croatian Roma groups, with the population size-weighted prevalence of the same star alleles in worldwide populations, grouped according to the ethnicity (listed in Supplementary Table S2 in the Gaedigk at el. 2016 paper). Alleles *10 and *41, predominantly found in Asian populations, are present in Croatian Roma with a substantially increased prevalence compared to their European average (Figure 4). The prevalence of diplotypes and their predicted phenotypes in the three groups of Croatian Roma are shown in Table 4. We found a total of 28 diplotypes, of which 14 define normal, 13 define intermediate and one diplotype—*4/*4—defines poor metabolizers. The most prevalent diplotypes in the Croatian Roma groups are *1/*4 diplotype, which defines intermediate metabolizers, and *1/*2 diplotype, which defines normal metabolizers—these two diplotypes were found in 28.3% of the total sample. They are followed by normal metabolizing *1/*41 diplotype and intermediate metabolizing *2/*4 diplotype prevalences. These four diplotypes were found in 46.5% of all Roma. On the other hand, 10 diplotypes were found only once in the total sample.

**Figure 5:**
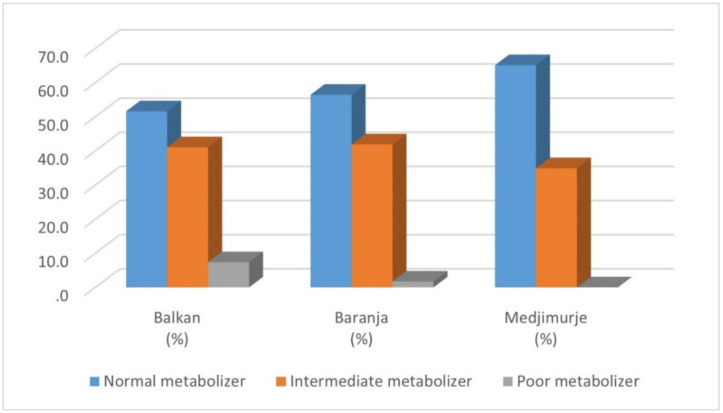
Distribution of estimated phenotype categories in three Roma groups.

The most prevalent phenotype was the normal metabolizing phenotype, found in 51.6% of Balkan Roma, 56.4% of Baranja Roma, and 65.1% of Medjimurje Roma. The intermediate metabolizing phenotype was found in 41.1% of Balkan Roma, 41.9% of Baranja Roma, and 34.9% of Medjimurje Roma. The poor metabolizing phenotype was found in 7.4% of the Balkan and 1.7% of the Baranja Roma, but it was not found in any sample from the Medjimurje Roma group (Figure 5). Altogether, the normal metabolizing phenotype was found in 57.9% of examinees, the intermediate metabolizing phenotype in 39.3%, and the poor metabolizing phenotype in 2.8% of the Croatian Roma.

## 4. Discussion

The Roma population is an example of founder populations, with centuries of sociocultural isolation. Due to the complex history of migration combined with the cultural practice of endogamy, the Roma appear as a structured group of populations [35,38]. Indeed, studies of mitochondrial DNA (mtDNA) showed a clear divergence of the Vlax Roma from the Balkan and other Roma groups that reached Europe as part of the first migration wave [50,51]. Similar results were found with autosomal [52] and Y STR loci [53]. All current research thus points to substantial differences in the genetic make-up of diverse Roma groups [54,55]. Due to the high influence of demographic history on the gene pool of the Roma, we were interested in finding out how it reflected SNP variations within the *CYP2D6* gene.

There were 43 polymorphic SNPs within the *CYP2D6* gene in the whole sample of Croatian Roma, some of which were considered almost monomorphic in the worldwide sample, and identified only in the gnomAD database [56]. One of these SNPs is the intron variant rs368389952, found only in the Balkan Roma with a MAF of 9.18%. Its frequency reported in the gnomAD database is 0.3% in South Asian populations and in the Uygur minority it has a frequency of 1% [57]. So far it has not been reported in the PharmVar database. The second intron variant, rs566383351, is also considered almost monomorphic in the world populations, but we found this polymorphism in Baranja (13.68%), Balkan (15.82%), and Medjimurje (10.65%) Roma, which is similar to the results of Ahmed (2018) who found this SNP’s minor allele in 14.59% of the Pakistani population [58]. This polymorphism has been reported in the PharmVar as a subvariant of star alleles CYP2D6*1, CYP2D6*35, and CYP2D6*41. It has also been reported in the Leiden Open Variation Database (LOVD) [59] as CYP2D6*35B with unknown effects. SNP rs374672076 is also considered monomorphic in the world population, but we have found it to be polymorphic in Balkan (8.67%) and Baranja (7.26%) Roma, which is again similar to Ahmed (2018), who found a frequency of 12.7% in the Pakistani population [58]. This SNP is an intron variant and it has been reported in the PharmVar as CYP2D6*139.001 with unknown function. SNP rs17002852, whose MAF in our total sample is <1%, is found among Baranja Roma with a frequency of 5.98%. The highest rs17002852 MAF of 2.56% is found among Middle Eastern populations (GnomAD). This synonymous variant has been reported in the PharmVar as subvariants CYP2D6*2.003, CYP2D6*2.007, CYP2D6*41.002, CYP2D6*131.001, and CYP2D6*149.001. Its association with tramadol response has been reported in the ClinVar [60].

For seven SNPs (rs4987144, rs28371730, rs28371725, rs16947, rs2267447, rs3892097, and rs1065852), we found similar frequencies in the Croatian Roma, South Asian, and European populations, which is not unexpected since the Roma originated in South Asia and began their migration to Europe more than a millennium ago.

A 89 distinct *CYP2D6* haplotypes of Croatian Roma were translated into the pharmacogenetically relevant star alleles. The least number of haplotypes were found among Roma from Medjimurje, which is in line with previous findings indicating that they are the least diverse Roma group in Croatia [51,53]. In our study, the most common star allele in all three Roma groups was the CYP2D6*1. It is considered a reference allele and makes up most of the star alleles in the European and African populations [1,61,62]. Of the three Croatian Roma groups, the lowest frequency of the reference allele *1 was observed among Balkan Roma. As shown in Figure 4, the Balkan Roma also have the lowest frequency of *1 when compared to many world populations. Star allele *2 was the second most common allele in the Balkan and Medjimurje Roma. Balkan and Medjimurje Roma groups have a similar prevalence of *2 as the European and South Asian populations, with Medjimurje frequency being closer to European and South Asian values. This is not surprising since Naveen (2006) showed that the distribution of CYP2D6*2 is similar between the European and South Asian populations [63]. The prevalence of CYP2D6*2 is about 28% among Europeans, 12–29% in the Asian populations, and 16–20% in people of African ancestry [33]. Sistonen (2007) proposed that long-term selective pressure maintains a high frequency of haplotypes encoding a fully functional enzyme, causing a homogeneous geographic distribution of *1 and *2 alleles [62].

The non-function CYP2D6*4 allele, which is predominantly found in European populations (18%), had the highest frequency in the Balkan and Baranja Roma groups, even higher than in European populations. The prevalence of the *4 allele in the Medjimurje Roma group is lower than in the European population but still higher than in other world populations. Our results are in concordance with those for Hungarian Roma, Czech Roma, and South Asians [64,65]. CYP2D6*4 creates a deficient protein [66] and contributes to most of the poor metabolizers observed in European populations. As a result of poor metabolization, a large accumulation of enzyme substrates occurs [67].

CYP2D6*10 is a decreased-function allele predominantly found in East and South Asian populations, where its prevalence ranges from 9% to 44%. Its frequency in African populations is between 4–6%, among Europeans < 2% [4,33], and it is also present in the Croatian Roma (6%). According to its prevalence, the Roma from Medjimurje (10%) are closest to the South Asians, especially South Indians [63], while the Balkan Roma (3%) are closer to European populations. This allele is considered an intermediate metabolizer phenotype, and individuals homozygous for this allele are at risk for adverse events, although not as severe as in poor metabolizers [68].

Finally, a decreased-function allele CYP2D6*41 is found in Croatian Roma at 14% frequency. Its prevalence among African populations is 4–11.5%, in Asian populations 2–12%, and about 9% in European populations [33]. Roma from Medjimurje (6%) are closer to European and South Asian populations, while the Balkan (17%) and Baranja Roma (18%) are closer to Middle East populations, which show the highest frequencies of *41 allele in the world (Figure 4).

Distribution of the five most frequent star alleles (*1, *2, *4, *10, and *41), which accounted for over 95% of the variance in all three Croatian Roma groups, did not significantly differ between Roma from Balkan and Baranja, while Roma from Medjimurje significantly differed from both these groups. The results on Roma from Medjimurje are in line with previous findings that showed the highest level of isolation compared to other Roma groups [51]. Although this research is missing the determination of structural variants, such as gene duplications, which are important for the accurate determination of phenotypes [37], we assessed the metabolizing phenotype from diplotype data. Distributions of metabolizing phenotypes are similar among Roma groups, but Roma from Medjimurje, with the highest frequency of normal metabolizers, the lowest frequency of intermediate metabolizers, and none of the poor metabolizers, are the most distinct. The results of Medjimurje Roma are similar to Hungarian Roma, which also had no poor metabolizers [64].

Because of their socio-economic status, the Roma have less access to medical care and are at higher risk of diseases like diabetes, cardiovascular diseases, and other complex diseases [69]. Since CYP2D6 metabolizes many commonly used drugs [70], it is of great interest for research not only in the general population but also in isolated or minority populations. Studies on clinical effects of several antiarrhythmic drugs including metoprolol, timolol and propafenone, and antidepressants and antipsychotics have not been unanimous. It is assumed that poor/intermediate metabolizers are prone to adverse drug reactions. Furthermore, for antidepressants and antipsychotics, there is a risk of overexposure in poor/intermediate metabolizers and underexposure in normal metabolizers [68]. For several opioid drugs (codeine, oxycodone, and tramadol) used to treat pain, genotypes have been shown to affect their efficacy and safety [68]. Cancer research studies are not in agreement with the role of CYP2D6 in the development of cancer [71,72]. Still, this enzyme is involved in the metabolism of cytotoxic drugs such as tamoxifen, and it has been shown that both poor and ultrarapid CYP2D6 metabolizers of tamoxifen have a worse prognosis compared with normal metabolizers [73,74].

Pharmacogenomic research is an important tool in drug development and health system improvement that leads to personalized medicine. In populations that are unlikely to have access to personalized medicine, a population profiling like this one can be of interest for medical practitioners since the results of such research can provide the basis for the avoidance (or careful monitoring of the effects) of the administration of drugs that contain substances that are not properly metabolized among the members of that population.

## 5. Conclusions

Summarizing our results, we can say that demographic history, predominantly migrations (from India to Balkans and across Southeast European areas) and endogamy, has indeed influenced the distribution of variations within the *CYP2D6* gene. It can be seen in the accumulation of globally rare variants which is the result of genetic drift that operates in isolated populations such as the Roma. Additionally, traces of their South Asian origin can be seen in the frequencies of polymorphic variants that are similar to Asian populations in many SNPs, as well as in elevated frequencies of star alleles *10 and *41. Given metabolizing phenotype estimates, Croatian Roma generally have low levels of poor metabolizers. The three socio-culturally different Roma groups studied differ significantly in the distribution of star alleles, which confirms the importance of studying different Roma groups separately.

## Figures and Tables

**Table 1:** Genotype and allele frequencies of *CYP2D6* polymorphisms in the three Croatian Roma samples (Balkan, Medjimurje, Baranja) and in the combined sample.

rsID	Clinical Implications	Genotypes and Alleles		Balkan *N* (%)	Medjimurje *N* (%)	Baranja *N* (%)	Combined *N* (%)	HWE Balkan	HWE Medjimurje	HWE Baranja	HWE CroRoma	X^2^	*p*
9200 G > C (rs1135840) missense variant	ultrarapid metabolism of debrisoquine, deutetrabenazine response, tamoxifen response, tramadol response, benign	genotype	G/G	8 (8.16%)	20 (18.52%)	14 (11.97%)	42 (13.00%)	0.8873	0.1119	0.6276	0.3772	18.22617	0.0991
			C/C	51 (52.04%)	44 (40.74%)	46 (39.32%)	141 (43.65%)						
			G/C	39 (39.80%)	43 (39.81%)	56 (47.86%)	138 (42.72%)						
		allele	C	141 (71.94%)	131 (61.21%)	148 (63.79%)	420 (65.42%)					5.62592	0.06003
8848 G > A (rs28371732) synonymous variant		genotype	G/G	96 (97.96%)	102 (94.44%)	92 (78.63%)	290 (89.78%)		0.9605		0.9766	1.83154	0.40021
			G/A	0	1 (0.93%)	0	1 (0.31%)						
		allele	A	0	1 (0.48%)	0	1 (0.17%)					1.82838	0.40084
8810 C > T (rs4987144) intron variant		genotype	C/C	44 (44.90%)	59 (54.63%)	59 (50.43%)	162 (50.15%)	0.8366	0.0538	0.066	0.0363	3.49495	0.47865
			T/T	10 (10.20%)	13 (12.04%)	16 (13.68%)	39 (12.07%)						
			C/T	44 (44.90%)	36 (33.33%)	42 (35.90%)	122 (37.77%)						
		allele	T	64 (32.65%)	62 (28.70%)	74 (31.62%)	200 (30.96%)					0.82556	0.66181
8604 G > A (rs28371730) intron variant		genotype	G/G	40 (40.82%)	45 (41.67%)	50 (42.74%)	135 (41.80%)	0.7361	0.8758	0.2036	0.3834	1.12704	0.88996
			A/A	14 (14.29%)	13 (12.04%)	19 (16.24%)	46 (14.24%)						
			G/A	44 (44.90%)	50 (46.30%)	48 (41.03%)	142 (43.96%)						
		allele	A	72 (36.73%)	76 (35.19%)	86 (36.75%)	234 (36.22%)					0.15128	0.92175
8602 A > G (rs2004511) intron variant		genotype	A/A	37 (37.76%)	30 (27.78%)	45 (38.46%)	112 (34.67%)	0.3336	0.0007	0.0088	**0.0001**	5.54417	0.23588
			G/G	11 (11.22%)	9 (8.33%)	7 (5.98%)	27 (8.36%)						
			A/G	50 (51.02%)	69 (63.89%)	65 (55.56%)	184 (56.97%)						
		allele	G	72 (36.73%)	87 (40.28%)	79 (33.76%)	238 (36.84%)					2.05158	0.35851
8565 dup (rs1269631565) intron variant		genotype	T/T	94 (95.92%)	86 (79.63%)	106 (90.60%)	286 (88.54%)	0.8366	0.2386	0.5936	0.2749	14.2025	**0.00082**
			TT/TT	0	0	0	0						
			T/TT	4 (4.08%)	22 (20.37%)	11 (9.40%)	37 (11.46%)						
		allele	TT	4 (2.04%)	22 (10.19%)	11 (4.70%)	37 (5.73%)					13.3396	0.00127
8504 G > A (rs867985262) intron variant		genotype	G/G	98 (100.00%)	107 (99.07%)	117 (100.00%)	322 (99.69%)		0.9614		0.9778	1.99692	0.36845
			G/A	0	1 (0.93%)	0	1 (0.31%)						
		allele	A	0	1 (0.46%)	0	1 (0.15%)					1.99383	0.36902
8498 A > G (rs79596243) intron variant		genotype	A/A	98 (100.00%)	107 (99.07%)	117 (100.00%)	322 (99.69%)		0.9614		0.9778	1.99692	0.36845
			A/G	0	1 (0.93%)	0	1 (0.31%)						
		allele	G	0	1 (0.46%)	0	1 (0.15%)					1.99383	0.36902
8455 C > A (rs28371729) intron variant	tramadol response	genotype	C/C	96 (97.96%)	107 (99.07%)	116 (99.15%)	319 (98.76%)	0.9187	0.9614	0.9630	0.9108	0.74298	0.68971
			C/A	2 (2.04%)	1 (0.93%)	1 (0.85%)	4 (1.24%)						
		allele	A	2 (1.02%)	1 (0.46%)	1 (0.43%)	4 (0.62%)					0.73835	0.69131
8413 T > C (rs28578778) intron variant		genotype	T/T	98 (100.00%)	108 (100.00%)	116 (99.15%)	322 (99.69%)			0.9630	0.9778	1.76615	0.41351
			T/C	0	0	1 (0.85%)	1 (0.31%)						
		allele	C	0	0	1 (0.43%)	1(0.15%)					1.76341	0.41408
8404 A > C (rs1985842) intron variant		genotype	A/A	8 (8.16%)	19 (17.59%)	13 (11.11%)	40 (12.38%)	1.000	0.2143	0.4040	0.7101	7.04036	0.13377
			C/C	50 (51.02%)	44 (40.74%)	46 (39.32%)	140 (43.34%)						
			A/C	40 (40.82%)	45 (41.67%)	58 (49.57%)	143 (44.27%)						
		allele	C	140 (71.43%)	133 (61.57%)	150 (64.10%)	423 (65.48%)					4.72262	0.0943
8199 C > T (rs200335621) synonymous variant		genotype	C/C	96 (97.96%)	106 (98.15%)	107 (91.45%)	309 (95.67%)	0.9187	0.9226	0.6292	0.6905	7.85586	0.01968
			C/T	2 (2.04%)	2 (1.85%)	10 (8.55%)	14 (4.33%)						
		allele	T	2 (1.02%)	2 (0.93%)	10 (4.27%)	14 (2.17%)					7.68184	0.02147
8180 G > C (rs141009491) missense variant		genotype	G/G	98 (100.00%)	108 (100.00%)	116 (99.15%)	322 (99.69%)			0.9630	0.9778	1.76615	0.41351
			G/C	0	0	1 (0.85%)	1 (0.31%)						
		allele	C	0	0	1 (0.43%)	1 (0.15%)					1.76341	0.41408
8008 G > A (rs28371725) intron variant	deutetrabenazine response, tamoxifen response, tramadol response	genotype	G/G	66 (67.35%)	88 (81.48%)	75 (64.10%)	229 (70.90%)	0.0543	0.4254	0.9517	0.1832	13.69378	0.00834
			A/A	5 (5.10%)	0 (0.00%)	4 (3.42%)	9 (2.79%)						
			G/A	20 (20.41%)	15 (13.89%)	34 (29.06%)	69 (21.36%)						
		allele	A	30 (16.48%)	15 (7.28%)	42 (18.58%)	87 (14.17%)					12.0599	0.00241
7870 C > T (rs16947) missense variant	benign, ultrarapid metabolism of debrisoquine, deutetrabenazine response, tamoxifen response, tramadol response	genotype	C/C	34 (34.69%)	46 (42.59%)	48 (41.03%)	128 (39.63%)	0.0511	0.6876	0.0393	0.0236	7.05147	0.13319
			T/T	21 (21.43%)	10 (9.26%)	21 (17.95%)	52 (16.10%)						
			C/T	35 (35.71%)	47 (43.52%)	42 (35.90%)	124 (38.39%)						
		allele	T	77 (42.78%)	67 (32.52%)	84 (37.84%)	228 (37.5%)					4.32612	0.11497
7632_7634 del (rs762158210) inframe deletion		genotype	GAGAA/ GAGAA	67 (68.37%)	37 (34.26%)	23 (19.66%)	127 (39.32%)	0.9028			0.9293	1.76652	0.41343
			GAGAA/ GA	2 (2.04%)	0	0	2 (0.62%)						
		allele	GA	2 (1.45%)	0	0	2 (0.78%)					1.75272	0.4163
7569 del (rs35742686) frameshift variant	poor metabolizer of debrisoquine	genotype	CAG/CAG	92 (93.88%)	97 (89.81%)	95 (81.20%)	284 (87.93%)	0.9584			0.9763	2.07179	0.35491
			CAG/CG	1 (1.02%)	0	0	1 (0.31%)						
		allele	CG	1 (0.54%)	0	0	1 (0.18%)					2.06814	0.3556
7503 G > T (rs28371717) missense variant	tramadol response	genotype	G/G	98 (100.00%)	108 (100.00%)	116 (99.15%)	322 (99.69%)			0.9630	0.9778	1.76615	0.41351
			G/T	0	0	1 (0.85%)	1 (0.31%)						
		allele	T	0	0	1 (0.43%)	1 (0.15%)					1.76341	0.41408
7490 T > C (rs17002852) synonymous variant	tramadol response	genotype	T/T	91 (92.86%)	108 (100.00%)	104 (88.89%)	303 (93.81%)	0.7139		0.3395	0.2439	12.93642	0.01159
			C/C	0	0	1 (0.85%)	1 (0.31%)						
			T/C	7 (7.14%)	0	12 (10.26%)	19 (5.88%)						
		allele	C	7 (3.57%)	0	14 (5.98%)	21 (3.25%)					12.87541	0.0016
7117 A > G (rs2267447) intron variant	tramadol response	genotype	A/A	53 (54.08%)	63 (58.33%)	63 (53.85%)	179 (55.42%)	0.4295	0.3927	0.2320	0.4934	3.98125	0.40855
			G/G	9 (9.18%)	4 (3.70%)	5 (4.27%)	18 (5.57%)						
			A/G	36 (36.73%)	41 (37.96%)	49 (41.88%)	126 (39.01%)						
		allele	G	54 (27.55%)	49 (22.68%)	59 (25.21%)	162 (25.08%)					2.08793	0.35206
6866 G > A (rs3892097) splice acceptor variant	amitriptyline response, antidepressants response—dosage. toxicity/ADR, clomipramine response, poor metabolizer of debrisoquine, deutetrabenazone response, tamoxifen response, tramadol response, desipramine response, doxepine response, imipramine response, nortriptyline response, trimipramine response, urinary metabolite levels in chronic kidney disease	genotype	G/G	57 (58.16%)	74 (68.52%)	71 (60.68%)	202 (62.54%)	0.5398	0.0522	0.0974	0.1578	11.76100	0.01922
			A/A	7 (7.14%)	0	2 (1.71%)	9 (2.79%)						
			G/A	34 (34.69%)	34 (31.48%)	44 (37.61%)	112 (34.67%)						
		allele	A	48 (24.49%)	34 (15.74%)	48 (20.51%)	130 (20.12%)					4.92789	0.0851
6769 A > G (rs1135824) missense variant	likely benign, germline origin	genotype	A/A	92 (93.88%)	108 (100.00%)	117 (100.00%)	317 (98.14%)	0.7546			0.8662	14.03625	**0.00090**
			A/G	6 (6.12%)	0	0	6 (1.86%)						
		allele	G	6 (3.06%)	0	0	6 (0.93%)					13.90466	**0.00096**
6684 C > T (rs1349481801) synonymous variant		genotype	C/C	98 (100.00%)	108 (100.00%)	116 (99.15%)	322 (99.69%)			0.9630	0.9778	1.76615	0.41351
			C/T	0	0	1 (0.85%)	1 (0.31%)						
		allele	T	0	0	1 (0.43%)	1 (0.15%)					1.76341	0.41408
6681 G > C (rs1058164) synonymous variant		genotype	G/G	8 (8.16%)	19 (17.59%)	13 (11.11%)	40 (12.38%)	1.000	0.2803	0.4040	0.7749	7.07838	0.13180
			C/C	50 (51.02%)	43 (39.81%)	46 (39.32%)	139 (43.03%)						
			G/C	40 (40.82%)	46 (42.59%)	58 (49.57%)	144 (44.58%)						
		allele	C	140 (71.43%)	132 (61.11%)	150 (64.10%)	422 (65.33%)					5.07114	0.0792
6460 T > C (rs376056664) intron variant		genotype	T/T	96 (97.96%)	105 (97.22%)	115 (98.29%)	316 (97.83%)	0.9593			0.9776	2.2752	0.3206
			C/C	0	0	0	0						
			T/C	1 (1.02%)	0	0	1 (0.31%)						
		allele	C	1 (0.52%)	0	0	1 (0.16%)					2.27162	0.32116
6313 G > A (rs189736703) intron variant		genotype	G/G	98 (100.00%)	108 (100.00%)	114 (97.44%)	320 (99.07%)			0.9626	0.9777	1.79690	0.40720
			G/A	0	0	1 (0.85%)	1 (0.31%)						
		allele	A	0	0	1 (0.43%)	1 (0.16%)					1.7941	0.40777
6188 G > A (rs1081004) intron variant	tramadol response	genotype	G/G	94 (95.92%)	103 (95.37%)	110 (94.02%)	307 (95.05%)	0.0001	**0.00002**	**<10^−5^**	**<10^−5^**	2.57173	0.63184
			A/A	1 (1.02%)	3 (2.78%)	5 (4.27%)	9 (2.79%)						
			G/A	3 (3.06%)	2 (1.85%)	2 (1.71%)	7 (2.17%)						
		allele	A	5 (2.56%)	8 (3.70%)	12 (5.13%)	25 (3.87%)					1.92838	0.38129
6089 G > A (rs368389952) intron variant		genotype	G/G	86 (87.76%)	108 (100.00%)	117 (100.00%)	311 (96.28%)	**<10^−5^**			**<10^−5^**	28.61408	**<10^−5^**
			A/A	6 (6.12%)	0	0	6 (1.86%)						
			G/A	6 (6.12%)	0	0	6 (1.86%)						
		allele	A	18 (9.18%)	0	0	18 (2.79%)					42.51105	**<10^−5^**
6057 C > T (rs1081003) synonymous variant		genotype	C/C	94 (95.92%)	96 (88.89%)	114 (97.44%)	304 (94.12%)	0.8366	0.5410	0.8883	0.5860	8.23424	0.01629
			C/T	4 (4.08%)	12 (11.11%)	3 (2.56%)	19 (5.88%)						
		allele	T	4 (2.04%)	12 (5.56%)	3 (1.29%)	19 (2.94%)					7.98471	0.01846
6015 C > G (rs28371705) synonymous variant		genotype	C/C	91 (92.86%)	108 (100.00%)	115 (98.29%)	314 (97.21%)	0.7139		0.9257	0.7996	10.4630	0.0054
			G/G	0	0	0	0						
			C/G	7 (7.14%)	0	2 (1.71%)	9 (2.79%)						
		allele	G	7 (3.57%)	0	2 (0.86%)	9 (1.39%)					10.31512	0.00576
6002 A > G (rs28371704) missense variant	tramadol response	genotype	A/A	91 (92.86%)	108 (100.00%)	115 (98.29%)	314 (97.21%)	0.7553		0.9257	0.8214	8.5260	0.0141
			G/G	0	0	0	0						
			A/G	6 (6.12%)	0	2 (1.71%)	8 (2.48%)						
		allele	G	6 (3.09%)	0	2 (0.86%)	8 (1.24%)					8.41879	0.01486
5992 C > A (rs28371703) intron variant		genotype	G/G	95 (96.94%)	108 (100.00%)	116 (99.15%)	319 (98.76%)			0.9630	0.9777	1.74048	0.41885
			G/A	0	0	1 (0.85%)	1 (0.31%)						
		allele	A	0	0	1 (0.43%)	1 (0.16%)					1.73776	0.41942
5289 C > T (rs29001678) noncoding transcript exon variant		genotype	C/C	80 (81.63%)	96 (88.89%)	97 (82.91%)	273 (84.52%)	0.9110	0.005	0.001	1.8 × 10^−5^	3.88908	0.42123
			T/T	0	1 (0.93%)	2 (1.71%)	3 (0.93%)						
			C/T	2 (2.04%)	0	2 (1.71%)	4 (1.24%)						
		allele	T	2 (1.22%)	2 (1.03%)	6 (2.97%)	10 (1.79%)					2.54616	0.27997
5264 A > G (rs1081000) noncoding transcript exon variant		genotype	A/A	86 (87.76%)	108 (100.00%)	115 (98.29%)	309 (95.67%)	0.5538		0.9257	0.7116	19.53312	**<10^−5^**
			A/G	11 (11.22%)	0	2 (1.71%)	13 (4.02%)						
		allele	G	11 (5.67%)	0	2 (0.85%)	13 (2.02%)					42.511	**<10^−5^**
5119 C > T (rs1065852) missense variant	poor metabolizer of debrisoquine, deutetrabenazone response, tamoxifen response, tramadol response, response to serotonin reuptake inhibitors in major depressive disorder	genotype	C/C	53 (54.08%)	63 (58.33%)	63 (53.85%)	179 (55.42%)	0.2532	0.6703	0.6483	0.8448	3.08176	0.54424
			T/T	10 (10.20%)	5 (4.63%)	7 (5.98%)	22 (6.81%)						
			C/T	35 (35.71%)	40 (37.04%)	47 (40.17%)	122 (37.77%)						
		allele	T	55 (28.06%)	50 (23.15%)	61 (26.07%)	166 (25.70%)					1.32564	0.5154
5101 C > T (rs138100349) missense variant		genotype	C/C	97 (98.98%)	106 (98.15%)	114 (97.44%)	317 (98.14%)	0.9595	0.9226	0.8883	0.8662	0.69712	0.70570
			C/T	1 (1.02%)	2 (1.85%)	3 (2.56%)	6 (1.86%)						
		allele	T	1 (0.51%)	2 (0.93%)	3 (1.28%)	6 (0.93%)					0.69059	0.70801
5050 G > A (rs769258) missense variant	tramadol response, likely benign	genotype	G/G	95 (96.94%)	108 (100.00%)	115 (98.29%)	318 (98.45%)	0.8777		0.9257	0.8885	3.19059	0.20285
			G/A	3 (3.06%)	0	2 (1.71%)	5 (1.55%)						
		allele	A	3 (1.53%)	0	2 (0.85%)	5 (0.77%)					3.1657	0.20539
4818 G > A (rs372204775) intron variant		genotype	G/G	95 (96.94%)	99 (91.67%)	111 (94.87%)	305 (94.43%)	0.9183	0.6514	0.7759	0.6266	4.02766	0.13348
			G/A	2 (2.04%)	9 (8.33%)	6 (5.13%)	17 (5.26%)						
		allele	A	2 (1.03%)	9 (4.17%)	6 (2.56%)	17 (2.64%)					3.91845	0.14097
4666 A > G (rs530422334)intron variant	tramadol response	genotype	A/A	98 (100.00%)	101 (93.52%)	113 (96.58%)	312 (96.59%)		0.7278	0.8508	0.7556	6.56138	0.03760
			A/G	0	7 (6.48%)	4 (3.42%)	11 (3.41%)						
		allele	G	0	7 (3.24%)	4 (1.71%)	11 (1.70%)					6.44772	0.0398
4655 G > A (rs1080992) intron variant		genotype	G/G	98 (100.00%)	106 (98.15%)	117 (100.00%)	321 (99.38%)		0.9226		0.9555	4.00629	0.13491
			G/A	0	2 (1.85%)	0	2 (0.62%)						
		allele	A	0	2 (0.93%)	0	2 (0.31%)					3.99385	0.13575
4623 G > T (rs769811346) intron variant		genotype	G/G	98 (100.00%)	108 (100.00%)	116 (99.15%)	322 (99.69%)			0.9630	0.9778	1.76615	0.41351
			G/T	0	0	1 (0.85%)	1 (0.31%)						
		allele	T	0	0	1 (0.43%)	1 (0.15%)					1.76341	0.41408
4622 G > C (rs374672076) intron variant		genotype	G/G	81 (82.65%)	98 (90.74%)	100 (85.47%)	279 (86.38%)	0.3471	0.6139	0.3968	0.1890	2.98457	0.22486
			G/C	17 (17.35%)	10 (9.26%)	17 (14.53%)	44 (13.62%)						
		allele	C	17 (8.67%)	10 (4.63%)	17 (7.26%)	44 (6.81%)					2.42978	0.29719
4589 C > T (rs566383351) intron variant		genotype	C/C	67 (68.37%)	85 (78.70%)	85 (72.65%)	237 (73.37%)	0.0629	0.2155	0.0866	0.0058	2.85917	0.23941
			C/T	31 (31.63%)	23 (21.30%)	32 (27.35%)	86 (26.63%)						
		allele	T	31 (15.82%)	23 (10.65%)	32 (13.68%)	86 (13.31%)					2.42008	0.29819

HWE (Bonferroni correction) *p* = 0.00037. X^2^ (Bonferroni correction) *p* = 0.0012. Significant values are shown in bold.

**Table 2:** Intra-population diversity and results of the Ewens–Watterson test of selective neutrality.

Roma Group	No. of Polym. Loci	No. of Haplotypes	Haplotype Diversity	Nucleotide Diversity	Observed F Value *	Expected F Value *	*p*-Value *
Balkan	27	46	0.9490	0.2046	0.0558	0.0546	0.6408
Baranja	26	47	0.9154	0.2035	0.0885	0.0574	0.9665
Medjimurje	21	37	0.9114	0.1791	0.0929	0.0762	0.8412

* Ewens–Watterson test of selective neutrality.

**Table 3:** Distribution of star alleles in the total Croatian Roma population and in the three subpopulations separately (Balkan, Baranja, Medjimurje).

Star Allele	Function ^†^	Balkan *N* (%)	Baranja *N* (%)	Medjimurje *N* (%)	Total *N* (%)
*1	normal	50 (25.91)	84 (35.90)	78 (36.45)	212 (33.07)
*2*	normal	49 (25.39)	42 (17.95)	58 (27.10)	149 (23.24)
*4*	no function	48 (24.87)	48 (20.51)	34 (15.89)	130 (20.28)
*10*	decreased	6 (3.11)	12 (5.13)	21 (9.81)	39 (6.08)
*22*	uncertain	1 (0.52)	0	2 (0.93)	3 (0.47)
*34*	normal	2 (1.04)	1 (0.43)	3 (1.40)	6 (0.94)
*35*	normal	2 (1.04)	2 (0.85)	0	4 (0.62)
*39*	uncertain	3 (1.55)	1 (0.43)	5 (2.34)	9 (1.40)
*41*	decreased	32 (16.58)	43 (18.38)	13 (6.07)	88 (13.73)
*65*	uncertain	0	1 (0.43)	0	1 (0.16)
Total		193 (100)	234 (100)	214 (100)	641 (100)

^†^ Function of star alleles was determined according to https://www.pharmvar.org/gene/CYP2D6, accessed on 18 January 2022.

**Table 4:** Distribution of star diplotypes and approximation of phenotypes in the total Croatian Roma population and in the three subpopulations separately (Balkan, Baranja, Medjimurje).

Star Diplotype	Phenotype	Balkan *N* (%)	Baranja *N* (%)	Medjimurje *N* (%)	Total *N* (%)
1/1	NM	6 (6.32)	14 (11.97)	17 (16.04)	37 (11,64)
1/2	NM	8 (8.42)	14 (11.97)	21 (19.81)	43 (13.52)
1/4	IM	16 (16.84)	22 (18.80)	9 (8.49)	47 (14.78)
1/10	NM	1 (1.05)	6 (5.13)	4 (3.77)	11 (3.46)
1/22	IM	1 (1.05)	0	1 (0.94)	2 (0.63)
1/34	NM	1 (1.05)	0	1 (0.94)	2 (0.63)
1/39	NM	1 (1.05)	0	2 (1.89)	3 (0.94)
1/41	NM	10 (10.53)	14 (11.97)	6 (5.66)	30 (9.43)
2/2	NM	10 (10.53)	4 (3.42)	8 (7.55)	22 (6.92)
2/4	IM	9 (9.47)	8 (6.84)	11 10.38)	28 (8.81)
2/10	NM	2 (2.10)	1 (0.85)	6 (5.66)	9 (2.83)
2/34	NM	0	0	1 (0.94)	1 (0.31)
2/35	NM	1 (1.05)	2 (1.71)	0	3 (0.94)
2/41	NM	8 (8.42)	9 (7.69)	3 (2.83)	20 (6.29)
4/4	PM	7 (7.37)	2 (1.71)	0	9 (2.83)
4/10	IM	2 (2.10)	5 (4.27)	9 (8.49)	16 (5.03)
4/34	IM	0	0	1 (0.94)	1 (0.31)
4/35	IM	1 (1.05)	0	0	1 (0.31)
4/39	IM	1 (1.05)	0	1 (0.94)	2 (0.63)
4/41	IM	4 (4.21)	9 (7.69)	3 (2.83)	16 (5.03)
10/10	IM	0	0	1 (0.94)	1 (0.31)
10/41	IM	1 (1.05)	0	0	1 (0.31)
22/41	IM	0	0	1 (0.94)	1 (0.31)
34/39	NM	1 (1.05)	0	0	1 (0.31)
34/41	NM	0	1 (0.85)	0	1 (0.31)
39/41	NM	0	1 (0.85)	0	1 (0.31)
41/41	IM	4 (4.21)	4 (3.42)	0	8 (2.52)
65/41	IM	0	1 (0.85)	0	1 (0.31)
Total		95 (100)	117 (100)	106 (100)	318 (100)

NM—normal metabolizer, IM—intermediate metabolizer, PM—poor metabolizer.

## Data Availability

All data analyzed in this study are available at http://roma.inantro.hr/en/, accessed on 18 January 2022. In case of using this database for further analyses, please cite this publication. If further clarification is required, contact the corresponding author.

## Supplementary Materials

The following supporting information can be downloaded at: https://www.mdpi.com/article/10.3390/jpm12030374/s1, Table S1: Major and minor allele frequencies of polymorphic loci in East Asian, South Asian, European, and Croatian Roma populations; Table S2: CYP2D6 haplotype list; Table S3: Worldwide frequencies of most frequent star alleles in Roma population.
